# Reply to Elliot Shevel

**DOI:** 10.1007/s10194-012-0435-8

**Published:** 2012-03-20

**Authors:** Pierangelo Geppetti, Eleonora Rossi, Alberto Chiarugi, Silvia Benemei

**Affiliations:** Headache Centre, Careggi University Hospital, University of Florence, Florence, Italy

In a recent review article we reconsidered the hypothesis that neurogenic vasodilatation is a key factor in the genesis of the headache of the migraine attack according to an updated and critical analysis of past and current literature. Cited papers span from pioneering studies in experimental animals of more than a century ago, to very recent investigations in humans in whom vasodilatation of cranial arteries has been accurately measured with highly sophisticated and reliable techniques. Results of neurovascular imaging studies have strongly corroborated previous pharmacological acquisition with antagonists of the calcitonin gene-related peptide receptor. Findings from clinical trials with these drugs underlined the role of neurogenic vasodilatation in migraine. In a comment to our review [1], Elliot Shevel [2] has appropriately noted that Zwetsloot et al. [3] and Schoonman et al. [4] indeed studied the middle cerebral artery and intracranial vessels and the last 10 mm of the external carotid artery. Dr. Shevel also noted that the papers by Ashina et al. [5] and Wienecke et al. [6] were performed in healthy volunteers in whom headache and not migraine-like pain was studied. Thus, all these findings do not negate the proposal by Graham and Woolf (sustained in our review article) that pertains to migraineurs and to the temporal artery (possibly limited to other extracranial arteries). Therefore, we thank Dr. Shevel for his observations that further support the view that vasodilatation brought about by activation of perivascular peptidergic nerve fibers should be considered as a major mechanism in migraine. This was, in fact, the main purpose of our review article.

On the other hand, we acknowledge that the complex pathophysiology of migraine and the mystery that still covers the initiating factors/mechanisms of the migraine attack should cast caution in refusing the contribution of triggers located in the central nervous system.
